# Nrdp1 increases neuron apoptosis via downregulation of Bruce following intracerebral haemorrhage

**DOI:** 10.1186/s12950-019-0229-8

**Published:** 2019-12-09

**Authors:** Changlong Zhou, Qingjun Liu, Wang Zhao, Ling Yang, Zhongyan Huang, Zhao Yang

**Affiliations:** 1 Department of Neurosurgery, Yongchuan Hospital, Chongqing Medical University, Chongqing, 402160 China; 2 Department of Neurology, Yongchuan Hospital, Chongqing Medical University, Chongqing, 402160 China

**Keywords:** Nrdp1, Neuron, Apoptosis, Bruce, ICH

## Abstract

**Background:**

Neuregulin receptor degradation protein-1 (Nrdp1) is an E3 ubiquitin ligase that plays an important role in regulating cell growth, apoptosis and oxidative stress. However, the data regarding its expression and exact mechanism in neuronal injury following ICH has not been well identified.

**Methods:**

In this study, primary cortical neurons from C57BL/6 mice were subjected to erythrocyte lysates. Nrdp1 expression, cell apoptosis, caspase-3 and BRUCE levels were detected. In addition, inflammatory response, brain edema, and neurological injury in ICH mice were also assessed.

**Results:**

We found that the expression of Nrdp1 was significantly increased in neuron cells accompanied by up-regulation of active caspase-3 and decreased expression of BRUCE (an inhibitor of apoptosis protein). However, inhibiting Nrdp1 levels of neurons reduced caspase-3 activity but induced up-regulation of BRUCE. In vivo, inhibiting Nrdp1 levels increased pro-inflammatory cytokines, brain edema, and neurological injury following ICH.

**Conclusions:**

Taken together, the data suggested that Nrdp1 might play a crucial role in neuronal apoptosis via inhibiting BRUCE following ICH.

## Background

Intracerebral hemorrhage (ICH) is the second largest type of stroke, which is associated with high mortality and morbidity [1–3]. Primary brain injury after ICH leads to hematoma effect and mechanical damage to adjacent brain tissues. Secondary brain injury is a key reason to cause nerve function damage following ICH [4–6]. Cell apoptosis is an important factor in secondary brain injury after ICH [4, 7, 8].

The ubiquitin-proteasome system (UPS) is the major intracellular machinery for protein degradation, which is responsible for maintaining cellular homeostasis by regulating cell apoptosis, cell division and cell signal transduction [9–11]. Neuregulin receptor degradation protein-1 (Nrdp1), a ring finger E3 ubiquitin ligase, plays an important role in regulating cell growth, apoptosis, oxidative stress and inflammation [12–14].

BRUCE/apollon is a huge membrane-associated protein containing one BIR domain at its N-terminal region [15]. It is exceptional in containing a C-terminal E2 motif, which can bind with Ub [16]. BRUCE has also been proposed to function as an E3, since some E3s can form bonds with Ub [17]. Recent evidence reports that Nrdp1 catalyzes ubiquitination and proteasomal degradation of BRUCE and promotes apoptosis [18].

However, the exact role of Nrdp1 in neuronal damage after ICH remains to be determined. In the present study, we tested role of Nrdp1 in primary cerebral neurons and ICH mice model. We observed substantial neuronal death and brain damage after ICH.

## Methods

### Animals

Eight week-old male specific pathogen-free (SPF) C57BL/6 mice were purchased from Chongqing Medical University and were housed in standard polypropylene cages at the animal facility until the day of the experiment. All procedures were performed in accordance with guidelines established by the Animal Care and Use Committee of Chongqing Medical University.

### Primary neuronal cell culture

Neuron-enriched cultures were prepared from brains of postnatal 24-h C57BL/6 mice. The meninges and blood vessels were removed from the brain and then brain tissues were digested with 0.25% trypsin (with EDTA) for 5 min at 37 °C. The tissues were washed three times with PBS to terminate trypsin digestion. Then, brain tissue suspensions were centrifuged at 1500 rpm. For 5 min, and the cells were suspended in a Neurobasal-A medium containing 2% B27, 2 m M L -glutamine, 50 U/ml penicillin and 50 U/ml streptomycin (all from Gibco, Carlsbad, CA, USA). Finally, cells were plated in 6 plates in a fresh medium and later half the medium was changed with fresh medium every 2 days. Purity of neuronal cultures was > 95% as confirmed by random staining with neuronal and glia markers (Tau or Iba1). Five days after plating, neuron had developed a dense network of extensions.

### Preparation of erythrocyte lysates

Spleens were removed from C57BL/6 mice. Single-cell suspensions of erythrocytes were prepared using stainless steel mesh screens. And then, 1 × 10^5^ erythrocytes were incubated with 1 ml red blood cell lysing solution for 20 min, and centrifuged at 2000 rpm for 10 min. The supernatants were utilized as erythrocyte lysates.

### ICH models in vitro

An ICH model in vitro was established by neuronal stimulation using erythrocyte lysates according to previous report [19]. Neurons (1 × 10^5^) were stimulated with 10 μl PBS or erythrocyte lysates for 48 h, and then cell medium was removed, washed three times with PBS and followed by other experiments.

### ICH model

ICH model in vivo was established by injection of autologous blood. After anesthesia with intraperitoneal injection of 4% chloral hydrate at a dosage of 1 ml/100 g. A 20-μl volume of autologous non-anti-coagulated blood was collected from the tail vein of the mouse and then injected into the caudate nucleus at 2 μl/min under stereotactic guidance at the following coordinates relative to bregma: 0.8 mm anterior, 2 mm left lateral, and 3.5 mm deep during a period of 10 min. The needle was held in place for 10 min after injection, and the microsyringe was pulled out after the blood had coagulated. Mice in the sham group were intracerebrally injected with 20 μl physiological saline solution. The bone hole was sealed with bone wax, and skin incision was disinfected and sutured. During the establishment of the model, body temperature was maintained at 37 °C throughout the procedure, and the mice were given free access to food and water after they woke up. The mice that died because of anesthesia were excluded.

### Tissue preparation

The brain was extracted and placed on ice. Using the needle track as the center, to prepare a coronal section and a sagittal section, the brain was cut and divided into four parts on the hematoma side: anterior-inner, anterior-outside, posterior-inner, and posterior-outside. From each of these quadrants, a total of 5 mm of brain tissue surrounding the hematoma was collected to further analysis.

### Western blot analysis

Western blot analysis was performed as indicated previously. Briefly, the brain samples or extracted cells were mechanically lysed in RIPA lysis buffer (Beyotime, Shanghai, China). Then we used enhanced BCA Protein Assay Kit (Beyotime) to measure protein concentrations by the bicinchoninic acid method. The protein samples (50 μg per lane) were then loaded onto a 10% SDS-polyacrylamide gel, separated and electrophoretically transferred to a polyvinylidene difluoride membrane (Millipore Corporation, Billerica, MA, USA), which was then blocked with 5% bovine serum albumin (BioSharp, Anhui, China) (1 h at room temperature). Then, the membrane was incubated for 12 h at 4 °C with.

primary antibodies against Nrdp1 (Santa Cruz, CA, USA), Bax/Bcl-2 (1:500; Santa Cruz), BRUCE (1:500; Santa Cruz) and β-actin (11,000; Santa Cruz). Later, the membrane was incubated with related HRP-conjugated secondary antibody (Santa Cruz Biotechnology) for 2 h at room temperature. We revealed the band signals via the Enhanced Chemiluminescence (ECL) Kit (Beyotime), and the relative quantity of proteins was analyzed via the Image J Software (NIH, Bethesda, MD, USA) and normalized to that of the loading control as discussed previously. In addition, the levels of phosphorylation were evaluated as the ratio of phosphoprotein to total protein.

### Real-time RT-PCR

Total RNA was isolated from neurons using Trizol reagents (Invitrogen Life Technologies, Carlsbad, CA, USA). RNA samples (2 μg) were reverse-transcribed to generate first-strand cDNA. After reverse transcription using TaqMan Reverse Transcription Kits (Applied Biosystems), reverse-transcribed products were amplified with the 7900HT real-time PCR System (Applied Biosystems) using SYBR Green PCR Master Mix (Applied Biosystems, Foster City, CA, USA) under the following conditions: 30 s at 95 °C, followed by a total of 40 cycles of two temperature cycles (15 s at 95 °C and 1 min at 60 °C). The Ct value was calculated by the comparative ^ΔΔ^CT method using the SDS Enterprise Database software (Applied Biosystems). The sequences of primers used were shown as following: Nrdp1, 5′-CCT GGC ATT TGA TGTTAC-3′ (forward), 5′-CAT GGG ATA TGA CTG CTC-3′ (reverse),

BRUCE, 5′-CTTGGTCTGAACACGAAAGACA-3′(forward), and 5′- TCCATCCGTACAAGGAAACTGT-3′(reverse), IL-6, 5′-AGCATACA GTTT GT GG ACATT-3′(forward), 5′-CAAC ATTCA TATTG CCA GTTCT − 3′(reverse); IL-1β, 5′- -CAGG CAACCAC TTACCT ATTTA − 3′ (forward), 5′-CCATA CACAC GGACAACAACTAGAT-3′ (reverse); TNF-α, 5′-CGAGTGACAAGCCTGTAGC-3′ (forward); 5′-TACTTGG GCAGATTGACCTCA-3′ (reverse). β-actin, 5′-GCAGCTCAGTAA CA GTCCGC-3′(forward), 5′-AGTGTGACGTTGACATCCGT-3′ (forward).

### Enzyme-linked immunosorbent assay

The perihematomal region of each ipsilateral hemisphere used for cytokine/chemokine quantification was homogenized and sonicated in RIPA buffer (Cell Signaling) with protease inhibitors (Phenylmethanesulfonyl fluoride), then centrifuged at 14,000×*g*. The protein concentration of the supernatant was determined using the BCA Protein Assay Kit (Thermo Fisher Scientific Inc.). One hundred microgram total protein was used for cytokine/chemokine quantification by multiplex ELISA (mouse inflammation panel I, Millipore) according to manufacturer’s instructions.

### MTT assay

Cell viability of neurone was assessed using 3-(4, 5- dimethyl-2- thiazolyl) -2,5-diphenyl-2H-tetrazolium bromide (MTT, Sigma-Aldrich) assay. After 48 h, MTT reagent was added to the wells, incubated at 37 °C for 4 h. After centrifugation, the supernatant was removed from each well. The coloured formazan crystal produced from MTT was dissolved with 0.15 ml DMSO, then the optical density (OD) value A490was measured by the multiscanner autoreader (Dynatech MR 5000; Dynatech Laboratories, Chantilly, VA, USA). The absorbance was measured at 570 nm. The mean of triplicate wells was taken as one value. The OD value for the control cultures was considered as 100% viability and viability in other samples is expressed as a percentage of viability in the control cultures.

### Annexin V and PI staining in vitro

After various treatments, neurons were trypsinized by 0.25% trypsin (without EDTA) and centrifuged at 1500 rpm for 5 min, and the resulting cell pellet was resuspended in 500 μl binding buffer. Later, 5 μl Annexin V and 5 μl PI (Beyotime, Shanghai, China) were added to the cell suspension. After 20 min of incubation at 37 °C in the dark, the cells were analyzed by flow cytometry (FACS Cabibur; BD, San Diego, CA, USA) and at least 20,000 events per sample were recorded.

### Caspase assay

To analyze caspase-3-like protease activities, the ApoAlert caspase-3 colorimetric assay kit (Clontech, Palo Alto, USA) was utilized. Cytosolic lysates were prepared 48 h following transfection and incubated with 50 mm p-nitroanilide (pNA) conjugated to the caspase cleavage site Asp-Glu-Val-Asp (DEVD) for 1 h at 37 °C. Hydrolyzed pNA was detected using a Multiscan MS colorimeter (Thermo Labsystems, Vantaa, Finland) at 405 nm. For control experiments, the lysates were incubated with 50 mM of the caspase-3 inhibitor DEVD-fmk (Clontech) for 30 min, before addition of the substrate.

### Intracerebroventricular injection

To investigate the effects of Nrdp1, Ad-control, Ad-Nrdp1 or Ad-si-Nrdp1 (2 μg/2 μl) was pretreated with a single intracerebroventricular (i.c.v.) injection in the ipsilateral ventricle 15 min before ICH. For the injection into the ipsilateral ventricle, a small burr hole was made in the parietal region (1.0 mm posterior and 1.0 mm lateral to the bregma) under the guidance of the stereotaxic instrument (RWD Life Science).

### Evaluation of neurological scores

The neurological scores were assessed by Neurological Severity Scores, according to the motor, sensory, reflex, and balance tests according to previous study [20]. Neurological function was assessed on a scale of 1–18; a score of 1 point is regarded as the inability to perform the test or for the lack of a tested reflex. The higher the score, the more severe the injury (normal score: 2–3; maximal deficit score: 18).

### Measurement of brain edema

Brain hemisphere were quickly separated and weighted to assess the wet weight (wW) using an electronic analytic balance. After drying the brain hemisphere in an oven at 100 °C for 24 h, dry tissue weight (dW) was assessed. The percentage of water was calculated according to the following formula: brain water content (%) = (wW-dW)/wW × 100%.

### Statistical analysis

Data are presented as mean ± SD. Statistical difference was determined by one-way ANOVA, followed by all pairwise multiple-comparison procedures with Bonferroni’s test. Bar chart values were analyzed by Student’s t-test. *P* < 0.05 was considered statistically significant. 

## Results

### Erythrocyte lysates induces Nrdp1 expression in neurons

**Fig. 1:**
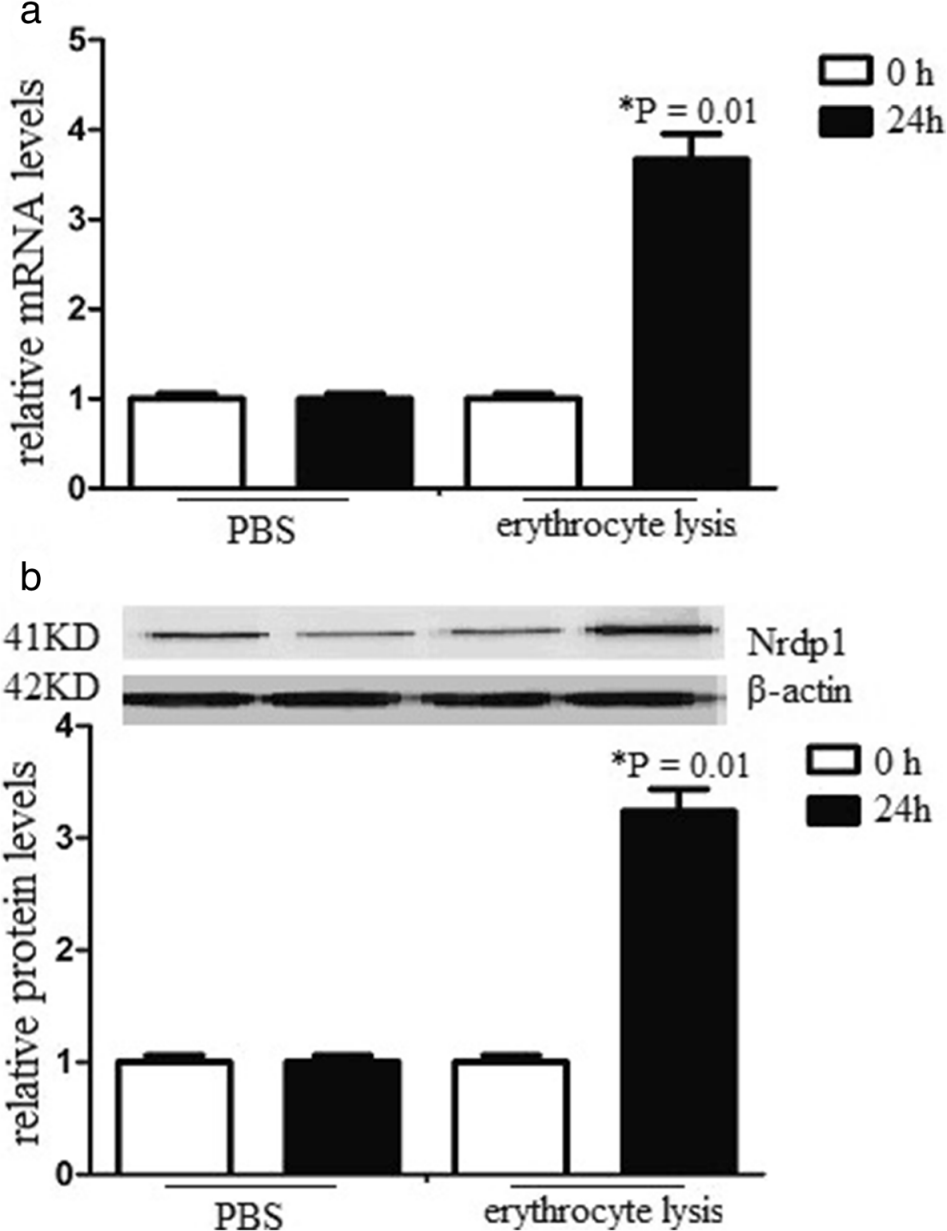
Erythrocyte lysates induces Nrdp1 expression in neurons. Neuron was isolated from the cerebral cortex of newborn mice, and subjected to erythrocyte lysis treatment for 0 or 24 h. **a** The Nrdp1 mRNA levels after erythrocyte lysis treatment was determined by real time RT-PCR. **b** The Nrdp1 protein levels after erythrocyte lysis treatment was determined by western blot analysis. Experiments performed in triplicate showed consistent results. Data are presented as the mean ± SD of three independent experiments. *P < 0.05

To determine the role of Nrdp1 in response to ICH, we examined the expression of Nrdp1 expression of neurons after erythrocyte lysates treatment. The cells were exposed to erythrocyte lysates for 0 or 24 h before analyzing Nrdp1 mRNA and protein levels. Real time RT-PCR and western blot analysis showed that Nrdp1 mRNA and protein levels increased in neurons after erythrocyte lysates treatment. However, there was no significant difference after PBS treatment (Fig. 1). The data suggested that erythrocyte lysates induced Nrdp1 expression in neurons.

### Nrdp1 promotes neuron apoptosis after erythrocyte lysates treatment

**Fig. 2:**
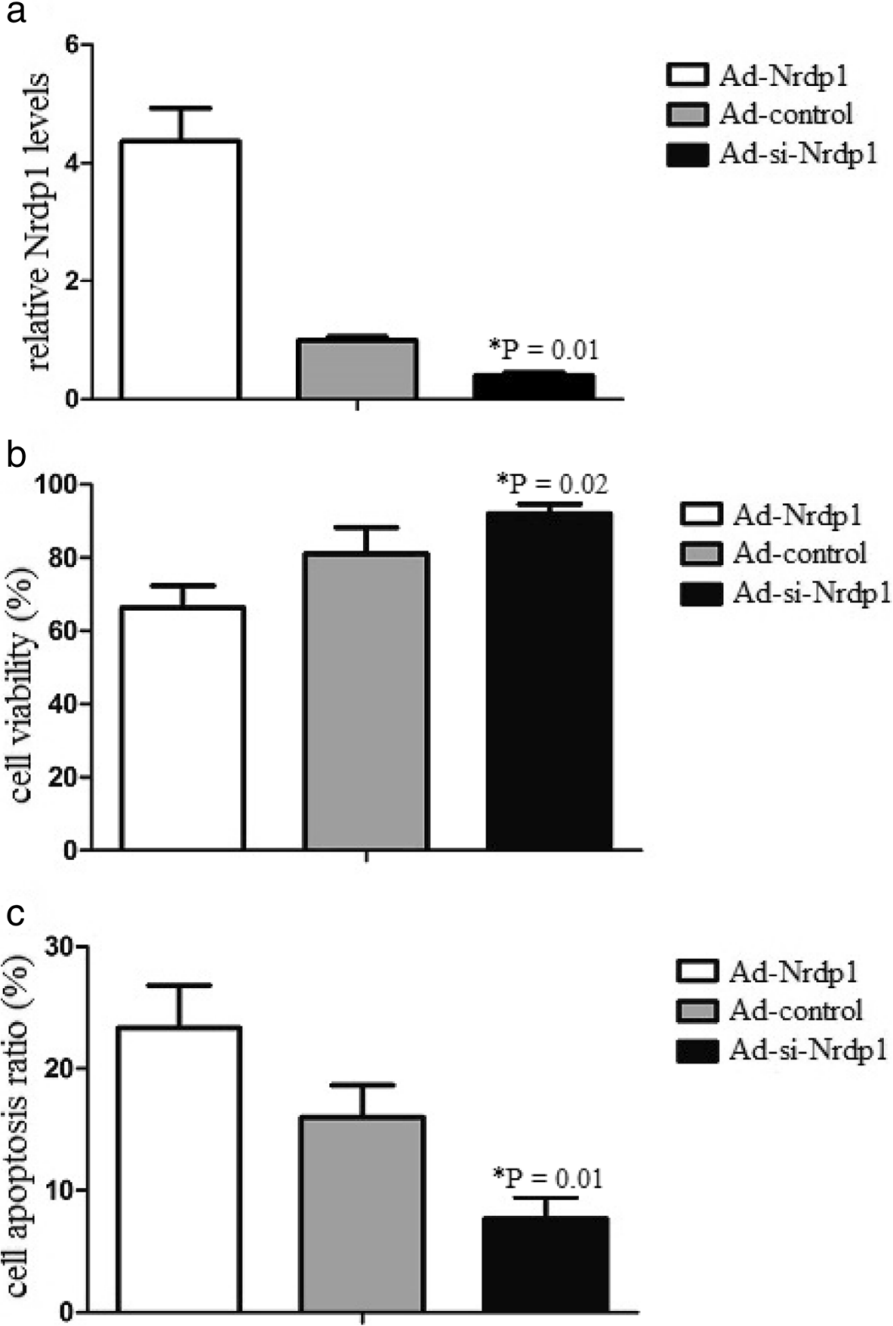
(**a**) Nrdp1 levels were detected by Western blot analysis. (**b**) MTT reagent was added and the cell viability was assessed. (**c**) Cell apoptosis ratio (%) was detected by flow cytometry. Apoptosis cells were determined by AnnexinV positive and propidium iodide (PI) negative cells

To identify whether Nrdp1 contributed to ICH-induced apoptosis in neurons, we transfected neurons with Ad-control, Ad-Nrdp1 or Ad-si-Nrdp1 before erythrocyte lysates treatment. Then, neurons were treated with erythrocyte lysates, and cell viability and cell apoptosis was detected by MTT and FACS assays. Western blot analysis showed that Ad-Nrdp1 significantly increased Nrdp1 levels, while Ad-si-Nrdp significantly decreased Nrdp1 levels (Fig. 2a). MTT data showed that Ad-Nrdp1 significantly decreased neuron viability, while Ad-si-Nrdp significantly increased neuron viability (Fig. 2b). FACS data showed that Ad-Nrdp1 significantly increased neuron apoptosis, while Ad-si-Nrdp significantly decreased neuron apoptosis (Fig. 2c). Therefore, the data suggested Nrdp1 promoted neuron apoptosis after erythrocyte lysates treatment.

### Nrdp1 promotes Bax/Bcl-2 ratio and caspase-3 activity

**Fig. 3:**
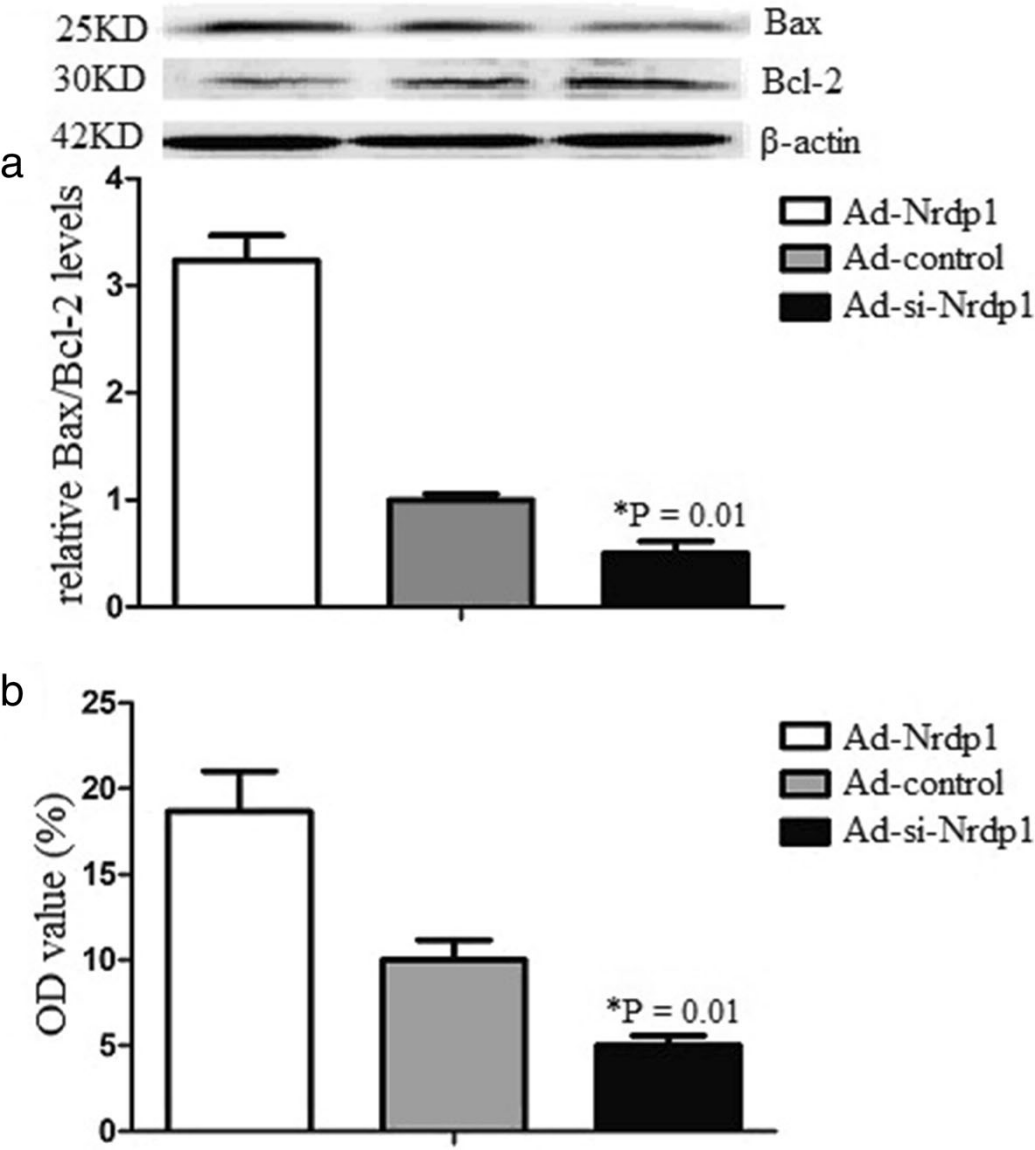
Nrdp1 promotes Bax/Bcl-2 ratio and caspase-3 activity. Neuron was isolated from the cerebral cortex of newborn mice. Ad-Nrdp1 or control transduced neurons were subjected to erythrocyte lysis treatment for 24 h. **a** Bax/Bcl-2 levels of neurons were determined by western blot analysis. **b** Caspase-3 activity was determined by the ApoAlert caspase-3 colorimetric assay kit. Hydrolysed pNA was detected using a Multiscan MS colorimeter at 405 nm

To further identify the specific mechanism of Nrdp1 in ICH-induced apoptosis in neurons, we assessed the several key apoptosis-associated signal proteins including Bax/Bcl-2 and caspase-3 activity. The results demonstrated that erythrocyte lysates promoted Bax/Bcl-2 ratio and caspase-3 activity of neurons. However, Ad-si-Nrdp1 attenuated Bax/Bcl-2 ratio and caspase-3 activity (Fig. 3). These results indicated that Nrdp1 played an important role in ICH-induced apoptosis of neurons.

### Nrdp1 inhibits BRUCE expression in vitro

**Fig. 4:**
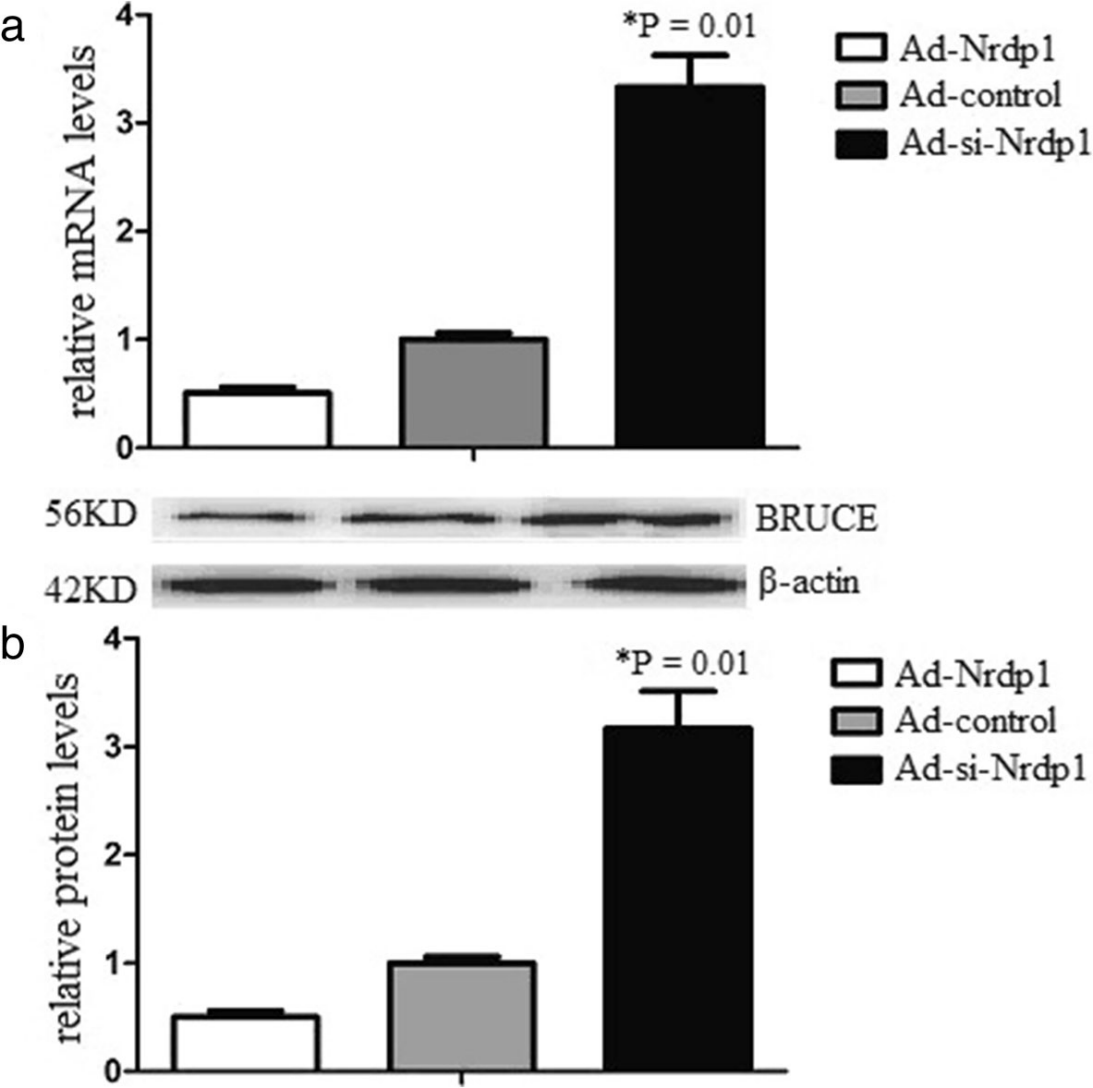
Nrdp1 inhibits BRUCE expression in vitro. Ad-Nrdp1 or control transduced neurons were subjected to erythrocyte lysis treatment for 24 h. **a**. The BRUCE mRNA levels were determined by real time RT-PCR. **b**. The BRUCE protein levels were determined by western blot analysis. Experiments performed in triplicate showed consistent results. Data are presented as the mean ± SD of three independent experiments. *P < 0.05

Related evidence demonstrated that the degradation of the inhibitor-of-apoptosis protein BRUCE/apollon is a key event in apoptosis. Moreover, much evidence indicates that Nrdp1 promotes ubiquitination and degradation of BRUCE in the pathology of numerous disorders. Therefore, we detected whether Nrdp1 could affect the expression of BRUCE in neurons. BRUCE levels of neurons were detected after erythrocyte lysates treatment. The results demonstrated that BRUCE was down-regulated after erythrocyte lysates treatment. However, and Ad-si-Nrdp1 increased BRUCE levels (Fig. 4). The findings suggested that Nrdp1 might promote neuronal apoptosis via inhibiting BRUCE levels.

### Nrdp1 increases brain inflammation after ICH

**Fig. 5:**
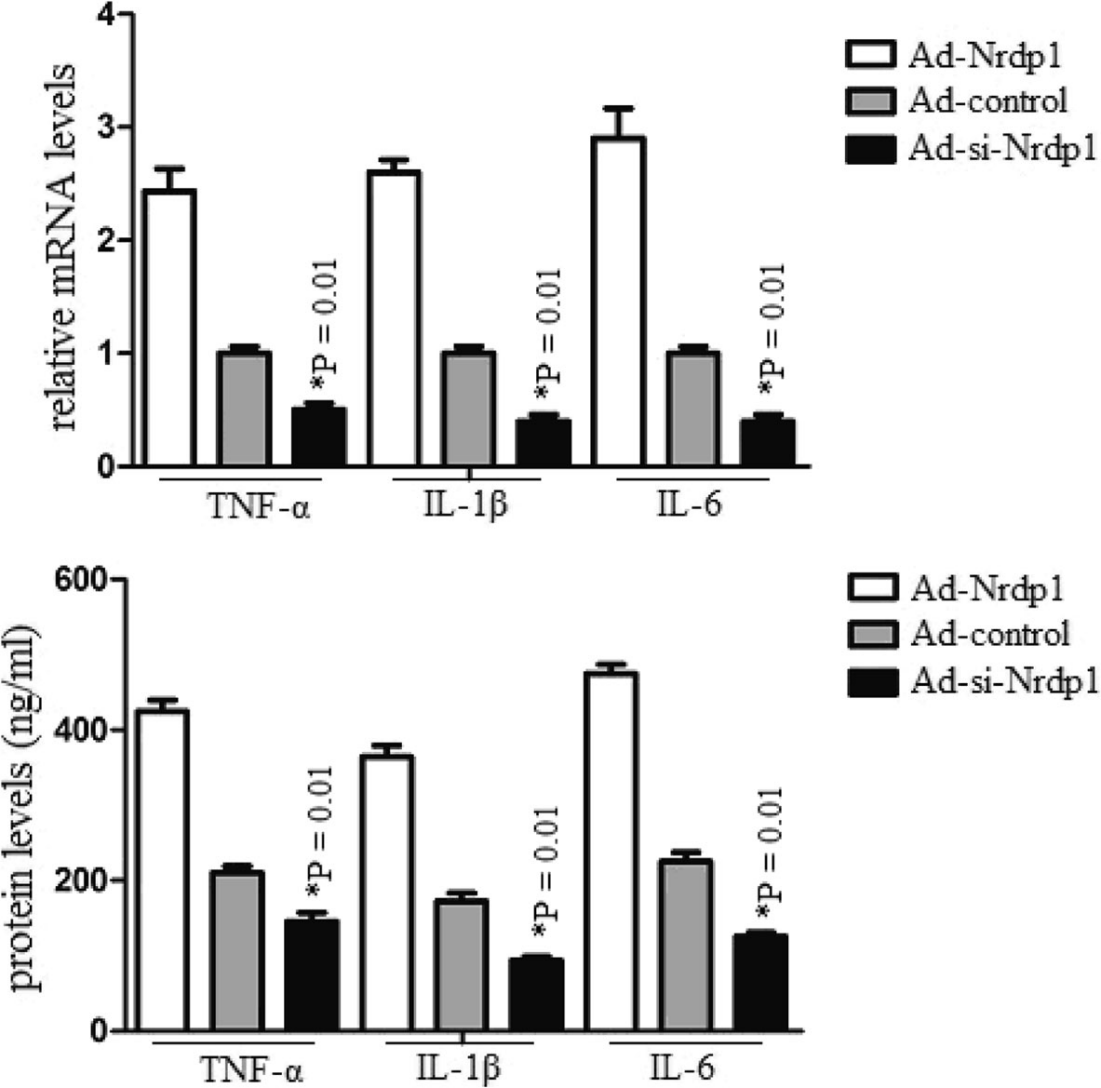
Nrdp1 increases brain inflammation after ICH. Intracerebroventricular injection of Ad-si-Nrdp1 or controls was administered 10 min after ICH. Mice were deeply anaesthetized and transcardially at 3 days after ICH. The brains were removed and post-fixed. The perihaematomal region of cerebral tissue was collected, inflammatory cytokines of the tissue lysates were further analyzed by qRT-PCR and western blot. Experiments performed in triplicate showed consistent results. Data are presented as the mean ± SD of three independent experiments. *P < 0.05

To determine the effect of Nrdp1 induced inflammation following ICH, we analyzed the levels of inflammatory cytokines in the hippocampus 3 days after ICH. The data showed that Ad-si-Nrdp1 significantly attenuated TNF-α, IL-1β and IL-6 levels in the hippocampus significantly after ICH. However, Ad-Nrdp1 significantly promoted TNF-α, IL-1β and IL-6 levels (Fig. 5). These results suggest that Nrdp1 promoted inflammation following ICH.

### Nrdp1 increases brain injury after ICH

**Fig. 6:**
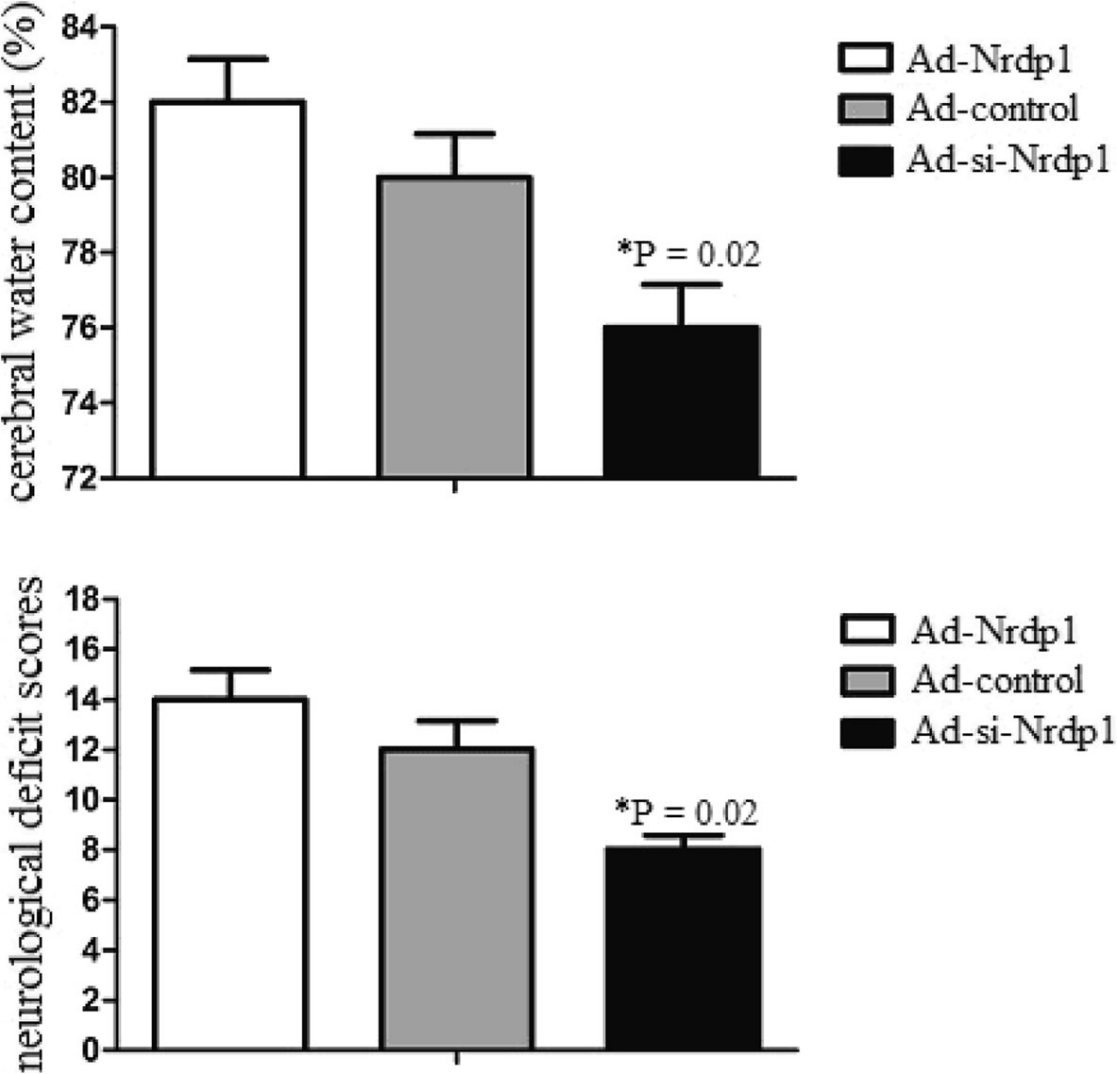
Nrdp1 increases brain injury after ICH. Intracerebroventricular injection of Ad-si-Nrdp1 or controls was administered 10 min after ICH. After 3 days of ICH, the cerebral water content of mice (*n* = 5) was also analyzed. In addition, the neurological deficit scores tests were performed by behavioral measurement, including a composite of motor, sensory, reflex, and balance tests. Experiments performed in triplicate showed consistent results. Data are presented as the mean ± SD of three independent experiments. *P < 0.05

In addition, to detect the role of Nrdp1 to brain injury, i.c.v. injection of Ad-control, Ad-Nrdp1 or Ad-si-Nrdp1 was administered 10 min after ICH. Water content and brain injury were observed 3 days after ICH. The results suggested that Ad-si-Nrdp1 significantly attenuated water content and brain injury. However, Ad-Nrdp1 significantly accelerated water content and brain injury (Fig. 6). The findings suggested that Nrdp1 increased brain injury after ICH.

## Discussion

In this study, our data demonstrate the following evidence: (1) erythrocyte lysates induces Nrdp1 expression in neurons; (2) Nrdp1 promotes neuron apoptosis after erythrocyte lysates treatment; (3) Nrdp1 promotes Bax/Bcl-2 ratio and caspase-3 activity; (4) Nrdp1 inhibits BRUCE expression in vitro; (5) Nrdp1 increases brain injury after ICH.

Neuroinflammation, which characterized as the activation of the innate immune system of the brain, plays a vital role in the pathogenesis of ICH [21–23]. In the course of neuroinflammation, microglia and astrocytes generate various cytokines, chemokines and pro-inflammatory factors, leading to neuronal apoptosis [24–26]. Therefore, neuronal apoptosis has been regarded as one of the most central points in ICH. Apoptosis is a highly regulated process and controlled by complex pro-apoptotic and anti-apoptotic factors. Initiation of classical apoptosis includes two main pathways-the intrinsic and the extrinsic pathway, eventually leading to caspase-3 activation [27–29]. The degradation of the inhibitor of apoptosis proteins (IAPs), such as BRUCE is another factor that involves in neuron death [30–32]. Although enormous studies have identified the pathology of neuroinflammatory process, the specific mechanism of neuronal apoptosis following ICH mediated inflammation has not fully reported.

Nrdp1 is a newly identified ring finger E3 ubiquitin ligase, and contributes to cell growth, apoptosis, and oxidative stress [33]. Related evidence demonstrated that up-regulation of Nrdp1 enhanced cardiac myocyte apoptosis and inhibition of endogenous Nrdp1 in cardiomyocytes protects against its apoptosis [34]. Neuronal apoptosis plays an important role in CNS inflammation [35–37]. Therefore, we explored whether Nrdp1 was involved in neuronal apoptosis following ICH.

In this study, we established ICH model in vitro and in vivo, and explored the role of Nrdp1 in the pathology of neuroinflammation.

Firstly, we found that erythrocyte lysates treatment induced Nrdp1 expression in neurons in primary neuronal cell culture. Secondly, we transfected neurons with Ad-Nrdp1 or contols, and detected neuron apoptosis. The data suggested Nrdp1 promoted neuron apoptosis via Bax/Bcl-2 ratio and caspase-3 activity after erythrocyte lysates treatment.

Related evidence demonstrated that the degradation of the gigantic (530 kDa) inhibitor-of-apoptosis protein BRUCE/apollon is a crucial point in apoptosis. BRUCE has also been identified to be degraded in neurons in the neurodegenerative diseases. In addition, Nrdp1 could promote caspase-3 activation and enhance cell apoptosis by reducing the expression of BRUCE. However, whether Nrdp1 could affect the expression of BRUCE in neurons after ICH has not been reported. Our data demonstrated that BRUCE was down-regulated after erythrocyte lysates treatment. However, upregulation of Nrdp1 increased BRUCE levels. The findings suggested that Nrdp1 promoted neuronal apoptosis via inhibiting BRUCE levels.

Lastly, to explore the potential role of Nrdp1 in inflammation and brain injury, we detected the levels of inflammatory cytokines, neurological severity scores and brain edema following ICH. The evidence demonstrated that Nrdp1 promoted inflammation and increased brain injury after ICH.

## Conclusions

In inclusion, our study suggested that Nrdp1 promoted inflammation and increased brain injury via inhibiting BRUCE after ICH. And the results indicated that Nrdp1 represented a promising therapeutical strategy in ICH.

## Data Availability

Please contact author for data requests.

## References

[1] Ovesen C, Christensen AF, Havsteen I, Krarup Hansen C, Rosenbaum S (2015). Prediction and prognostication of neurological deterioration in patients with acute ICH: a hospital-based cohort study. BMJ Open.

[2] Meyer DM, Begtrup K, Grotta JC (2015). Is the ICH score a valid predictor of mortality in intracerebral hemorrhage?. J Am Assoc Nurse Pract.

[3] Mustanoja S, Satopaa J, Meretoja A, Putaala J, Strbian D (2015). Extent of secondary intraventricular hemorrhage is an independent predictor of outcomes in intracerebral hemorrhage: data from the Helsinki ICH study. Int J Stroke.

[4] He M, Wang Y, Shen J, Duan C, Lu X (2018). Bex1 attenuates neuronal apoptosis in rat intracerebral hemorrhage model. Pathol Res Pract.

[5] Meng C, Zhang J, Dang B, Li H, Shen H (2018). PERK pathway activation promotes Intracerebral hemorrhage induced secondary brain injury by inducing neuronal apoptosis both in vivo and in vitro. Front Neurosci.

[6] Wang Zhifeng, Chen Zhouqing, Yang Junjie, Yang Ziying, Yin Jia, Duan Xiaochun, Shen Haitao, Li Haiying, Wang Zhong, Chen Gang (2018). Treatment of secondary brain injury by perturbing postsynaptic density protein-95-NMDA receptor interaction after intracerebral hemorrhage in rats. Journal of Cerebral Blood Flow & Metabolism.

[7] Zhou L, Liu C, Wang Z, Shen H, Wen Z (2018). Pannexin-1 is involved in neuronal apoptosis and degeneration in experimental intracerebral hemorrhage in rats. Mol Med Rep.

[8] Wang J, Zhai W, Yu Z, Sun L, Li H (2017). Neuroprotection exerted by Netrin-1 and Kinesin motor KIF1A in secondary brain injury following experimental Intracerebral hemorrhage in rats. Front Cell Neurosci.

[9] Murdoch JD, Rostosky CM, Gowrisankaran S, Arora AS, Soukup SF (2016). Endophilin-a deficiency induces the Foxo3a-Fbxo32 network in the brain and causes Dysregulation of autophagy and the ubiquitin-proteasome system. Cell Rep.

[10] Yu P, Fan Y, Qu X, Zhang J, Song N (2016). Cbl-b regulates the sensitivity of cetuximab through ubiquitin-proteasome system in human gastric cancer cells. J Buon.

[11] Li X, Zhu F, Jiang J, Sun C, Zhong Q (2016). Simultaneous inhibition of the ubiquitin-proteasome system and autophagy enhances apoptosis induced by ER stress aggravators in human pancreatic cancer cells. Autophagy.

[12] Zhang DL, Han F, Yu DH, Xiao SJ, Li MY (2015). Characterization of E3 ubiquitin ligase neuregulin receptor degradation protein-1 (Nrdp1) in the large yellow croaker (Larimichthys crocea) and its immune responses to Cryptocaryon irritans. Gene.

[13] Printsev I, Yen L, Sweeney C, Carraway KL (2014). Oligomerization of the Nrdp1 E3 ubiquitin ligase is necessary for efficient autoubiquitination but not ErbB3 ubiquitination. J Biol Chem.

[14] Lewandowski KT, Piwnica-Worms H (2014). Phosphorylation of the E3 ubiquitin ligase RNF41 by the kinase par-1b is required for epithelial cell polarity. J Cell Sci.

[15] Qiu XB, Markant SL, Yuan J, Goldberg AL (2004). Nrdp1-mediated degradation of the gigantic IAP, BRUCE, is a novel pathway for triggering apoptosis. EMBO J.

[16] Qiu XB, Goldberg AL (2005). The membrane-associated inhibitor of apoptosis protein, BRUCE/Apollon, antagonizes both the precursor and mature forms of Smac and caspase-9. J Biol Chem.

[17] Ren J, Shi M, Liu R, Yang QH, Johnson T (2005). The Birc6 (Bruce) gene regulates p53 and the mitochondrial pathway of apoptosis and is essential for mouse embryonic development. Proc Natl Acad Sci U S A.

[18] Shen J, Song Y, Lin Y, Wu X, Yan Y (2015). Nrdp1 is associated with neuronal apoptosis in lipopolysaccharide-induced Neuroinflammation. Neurochem Res.

[19] Wang Z, Fang L, Shi H, Yang Z (2019). miR-181b regulates ER stress induced neuron death through targeting heat shock protein A5 following intracerebral haemorrhage. Immunol Lett.

[20] Wang YC, Wang PF, Fang H, Chen J, Xiong XY (2013). Toll-like receptor 4 antagonist attenuates intracerebral hemorrhage-induced brain injury. Stroke.

[21] Ren H, Kong Y, Liu Z, Zang D, Yang X (2018). Selective NLRP3 (Pyrin domain-containing protein 3) Inflammasome inhibitor reduces brain injury after Intracerebral hemorrhage. Stroke.

[22] Xu C, Wang T, Cheng S, Liu Y (2013). Increased expression of T cell immunoglobulin and mucin domain 3 aggravates brain inflammation via regulation of the function of microglia/macrophages after intracerebral hemorrhage in mice. J Neuroinflammation.

[23] Fang H, Wang PF, Zhou Y, Wang YC, Yang QW (2013). Toll-like receptor 4 signaling in intracerebral hemorrhage-induced inflammation and injury. J Neuroinflammation.

[24] Choi KS, Kim HJ, Do SH, Hwang SJ, Yi HJ (2018). Neuroprotective effects of hydrogen inhalation in an experimental rat intracerebral hemorrhage model. Brain Res Bull.

[25] Wei N, Wei Y, Li B, Pang L (2017). Baicalein promotes neuronal and behavioral recovery after Intracerebral hemorrhage via suppressing apoptosis, oxidative stress and Neuroinflammation. Neurochem Res.

[26] Lei C, Lin S, Zhang C, Tao W, Dong W (2013). High-mobility group box 1 protein promotes neuroinflammation after intracerebral hemorrhage in rats. Neuroscience.

[27] Juraver-Geslin HA, Durand BC (2015). Early development of the neural plate: new roles for apoptosis and for one of its main effectors caspase-3. Genesis.

[28] Tian H, Zhang DF, Zhang BF, Li HZ, Zhang Q (2015). Melanoma differentiation associated gene-7/interleukin-24 induces caspase-3 denitrosylation to facilitate the activation of cancer cell apoptosis. J Interf Cytokine Res.

[29] Loison F, Xu Y, Luo HR (2014). Proteinase 3 and Serpin B1: a novel pathway in the regulation of caspase-3 activation, neutrophil spontaneous apoptosis, and inflammation. Inflamm Cell Signal.

[30] Chen SJ, Lin JH, Yao XD, Peng B, Xu YF (2016). Nrdp1-mediated degradation of BRUCE decreases cell viability and induces apoptosis in human 786-O renal cell carcinoma cells. Exp Ther Med.

[31] Jaquith JB (2014). Targeting the inhibitor of apoptosis protein BIR3 binding domains. Pharm Pat Anal.

[32] Domingues C, Ryoo HD (2012). Drosophila BRUCE inhibits apoptosis through non-lysine ubiquitination of the IAP-antagonist REAPER. Cell Death Differ.

[33] Zhou A, Pan D, Yang X, Zhou J (2011). Overexpression of Nrdp1/FLRF sensitizes cells to oxidative stress. Biochem Biophys Res Commun.

[34] Zhang Y, Kang YM, Tian C, Zeng Y, Jia LX (2011). Overexpression of Nrdp1 in the heart exacerbates doxorubicin-induced cardiac dysfunction in mice. PLoS One.

[35] Chen J, Wang Z, Zheng Z, Chen Y, Khor S (2017). Neuron and microglia/macrophage-derived FGF10 activate neuronal FGFR2/PI3K/Akt signaling and inhibit microglia/macrophages TLR4/NF-kappaB-dependent neuroinflammation to improve functional recovery after spinal cord injury. Cell Death Dis.

[36] Marwarha G, Ghribi O (2017). Nuclear factor kappa-light-chain-enhancer of activated B cells (NF-kappaB) - a friend, a foe, or a bystander - in the neurodegenerative Cascade and pathogenesis of Alzheimer's disease. CNS Neurol Disord Drug Targets.

[37] Ramesh G, Martinez AN, Martin DS, Philipp MT (2017). Effects of dexamethasone and meloxicam on Borrelia burgdorferi-induced inflammation in glial and neuronal cells of the central nervous system. J Neuroinflammation.

